# Bifurcation treatment with novel, highly flexible drug-eluting coronary stents in all-comers: 2-year outcome in patients of the DUTCH PEERS trial

**DOI:** 10.1007/s00392-015-0907-3

**Published:** 2015-09-02

**Authors:** Liefke C. van der Heijden, Marlies M. Kok, Ming Kai Lam, Peter W. Danse, Alexander R. Schramm, Gillian A. J. Jessurun, R. Melvyn Tjon Joe Gin, K. Gert van Houwelingen, Raymond W. M. Hautvast, Gerard C. M. Linssen, Hanim Sen, Marije M. Löwik, Maarten J. IJzerman, Carine J. M. Doggen, Clemens von Birgelen

**Affiliations:** Department of Cardiology, Thoraxcentrum Twente, Medisch Spectrum Twente, Haaksbergerstraat 55, 7513 ER Enschede, The Netherlands; Department of Cardiology, Rijnstate Hospital, Arnhem, The Netherlands; Department of Cardiology, Scheper Hospital, Emmen, The Netherlands; Department of Cardiology, Medisch Centrum Alkmaar, Alkmaar, The Netherlands; Department of Cardiology, Ziekenhuisgroep Twente, Almelo, The Netherlands; Department of Cardiology, Ziekenhuisgroep Twente, Hengelo, The Netherlands; Department Health Technology and Services Research, MIRA-Institute for Biomedical Technology and Technical Medicine, University of Twente, Enschede, The Netherlands

**Keywords:** Bifurcation/coronary bifurcation, Drug-eluting stents (DES), Percutaneous coronary intervention (PCI), DUTCH PEERS (TWENTE II) randomized clinical trial, Promus element platinum–chromium everolimus-eluting stent (PE-PtCr-EES), Resolute integrity cobalt–chromium zotarolimus-eluting stent (RI-CoCr-ZES)

## Abstract

**Background:**

Percutaneous coronary intervention (PCI) in bifurcated lesions with second-generation drug-eluting stents (DES) was associated with increased myocardial infarction (MI) rates. Flexible stent designs that accommodate well to vessel tapering may be of benefit in challenging anatomies such as bifurcated target lesions, but so far data are scarce.

**Methods:**

We analyzed the 2-year follow-up data of the DUTCH PEERS (TWENTE II) trial, which randomized 1811 all-comer patients to PCI with newer generation resolute integrity zotarolimus-eluting (Medtronic) or promus element everolimus-eluting stents (Boston Scientific). In bifurcated lesions, provisional stenting was generally performed. Target vessel failure is a composite endpoint, consisting of cardiac death, target vessel MI, or target vessel revascularization.

**Results:**

Patients with at least one bifurcated lesion (*n* = 465, 25.7 %) versus patients with non-bifurcated target lesions only (*n* = 1346, 74.3 %) showed similar rates of clinical endpoints including target vessel failure (9.2 versus 7.9 %, *p* = 0.36) and definite stent thrombosis (0.4 versus 1.0 %, *p* = 0.38). Target vessel MI was more common in patients with bifurcated lesions (3.4 versus 1.6 %, *p* = 0.02); but after multivariate analysis with propensity score adjustment, bifurcation treatment was found not to be an independent predictor of target vessel MI (HR 1.40, 95 % CI 0.71–2.76; *p* = 0.34). Among patients with bifurcated lesions, DES type and side-branch size did not affect outcome, but periprocedural MI occurred more often after two-stent approaches (9.0 versus 2.1 %; *p* = 0.002).

**Conclusion:**

All-comer patients treated for bifurcated and non-bifurcated target lesions showed similar and low rates of clinical endpoints, suggesting that the DES used are efficacious and safe for treating bifurcated target lesions.

## Introduction

Stenting of bifurcated target lesions is among the most challenging procedures in the field of percutaneous coronary intervention (PCI) [1, 2]. In bifurcated lesions, the introduction of the first generation of drug-eluting stents (DES) reduced the need for repeat revascularization as compared to the previously used bare metal stents [2–8]. DES of the second generation, which employed coatings with an improved biocompatibility and thinner struts than the first generation, have shown favorable clinical results in both broad patient populations [9–18] and bifurcated lesions [19–21]. Despite generally encouraging clinical outcomes, the rate of myocardial infarction (MI), in particular of periprocedural MI (PMI), was still higher following stenting of bifurcated lesions as compared to non-bifurcated lesions [20, 21].

Recently, novel DES have been developed with thinner struts and/or more flexible stent designs that accommodate well to vessel tapering, which may be of benefit in challenging anatomies such as bifurcated lesions [22, 23]. The DUTCH PEERS randomized trial compares two such DES in an all-comer patient population and has shown similar and favorable results for both devices up to 2-year follow-up in the overall study population [24, 25].

While the use of highly flexible DES has resulted in an overall low MI rate [24], it is unknown whether the implantation of such modern stents may still be associated with an increased risk of MI in bifurcated target lesions. In the present study, we assessed the hypothesis that there may be no difference in safety and efficacy of these flexible DES in treating patients with bifurcated versus non-bifurcated target lesions. In addition, among patients with bifurcated lesions, we evaluated the potential impact of stent type, side-branch size, kissing-balloon inflation, and technical complexity of the procedure on 2-year clinical outcome.

## Methods

### Patient population and study design

The present study was performed using the 2-year follow-up data of the randomized, patient-blinded, multicenter DUTCH PEERS trial [25]. Details of the DUTCH PEERS (TWENTE II) trial (ClinicalTrials.gov NCT01331707) have previously been reported [24]. In brief, the trial compares (1:1 randomization) the resolute integrity zotarolimus-eluting cobalt–chromium stent (Medtronic Vascular, Santa Rosa, CA) and the promus element everolimus-eluting platinum–chromium stent (Boston Scientific, Natick, MA) in 1811 all-comer patients. Patients were enrolled between November 25, 2010 and May 24, 2012. The trial complies with the Declaration of Helsinki and was approved by the Medical Ethics Committee Twente and the institutional review boards of all participating centers. All patients provided written informed consent. Interventional procedures and application of concomitant medication were performed in accordance to medical guidelines, clinical standards, and the physician’s judgment. The generally recommended approach of bifurcation lesion treatment was provisional stenting, but the technique of stenting, medical treatment strategy, and use of final kissing-balloon inflation were left at the operator’s discretion [24].

### Clinical follow-up, monitoring, adjudication, and angiographic analysis

A detailed description of the 2-year follow-up data has previously been reported [25]. Data monitoring was performed by the independent contract research organization (CRO) Diagram (Zwolle, the Netherlands). The independent CRO Cardialysis (Rotterdam, the Netherlands) performed the processing of clinical outcome data and clinical event adjudication. Experienced angiographic analysts from Thoraxcentrum Twente, blinded for the stent type and clinical outcome, performed offline quantitative coronary angiographic analyses according to current standards for all patients from the four study centers (Qangio XA 7.2, Medis, Leiden, the Netherlands).

### Data analysis

For the purpose of the present analysis, patients were categorized into patients with at least one bifurcated target lesion versus patients with non-bifurcated lesions. A relevant side-branch was defined, according to the definition in the SYNTAX score, as a junction of a main vessel and a side-branch with minimum lumen diameter ≥1.5 mm (after intracoronary administration of nitrates and before PCI), as measured by quantitative coronary angiography [26]. Further analyses among patients with bifurcated lesions involved comparisons between (1) the two allocated stents; (2) bifurcated lesions with side-branch ≥2.0 mm versus side-branch <2.0 mm, as measured by quantitative coronary angiography; (3) the use of final kissing-balloon inflation versus no final kissing; and (4) single versus two-stent approach.

### Clinical endpoints

Clinical endpoints were defined according to the Academic Research Consortium (ARC), including the addendum on myocardial infarction [27, 28]. Death was considered cardiac, unless an evident non-cardiac cause could be established. Myocardial infarction (MI) was defined by any creatine kinase concentration of more than double the upper limit of normal with elevated values of a confirmatory cardiac biomarker. PMI was defined as target vessel MI within 48 h after PCI. Stent thrombosis was classified according to the ARC definitions. The composite endpoint target vessel failure was defined as cardiac death, target vessel MI, or clinically driven target vessel revascularization. Target lesion failure was defined as a composite of cardiac death, target vessel MI, and clinically indicated target lesion revascularization. A patient-oriented composite endpoint consisted of all-cause death, any MI, and any repeat revascularization. Major adverse cardiac events were classified as a composite of all-cause death, any MI, emergent coronary artery bypass grafting, and clinically indicated target lesion revascularization.

### Statistical analysis

Data were reported as frequencies and percentages for dichotomous and categorical variables, as mean ± standard deviation for continuous normally distributed variables and as median and inter-quartile range for not normally distributed variables. Chi-square test and Fisher’s exact test were used as appropriate. Differences between groups in continuous variables were assessed with the Student’s *t* test or the Wilcoxon rank-sum test. The Kaplan–Meier method was used to calculate the time to clinical endpoint and the Log-rank test was applied to compare groups. Parameters were considered as potential confounders if associations were found with a *p* value ≤0.15 in univariate analyses. For adjustment of potential confounders, propensity score analysis was used. The propensity score was estimated using multiple logistic regression analysis. Gender, clinical syndrome, RCA treatment, LAD treatment, multivessel disease, small vessel treatment, total stent length, number of stents per patient, postdilation, and lesion length longer than 27 mm were used to calculate the propensity score for having a bifurcated target lesion. A multivariate Cox regression model, including the propensity score as independent variable, was then used to adjust for the propensity score. All *p* values and confidence intervals were two-sided and *p* values <0.05 were considered significant. Data analysis was performed with SPSS (version 22.0, SPSS Inc., Chicago, IL, USA).

## Results

### Baseline, lesion, and procedural characteristics

Of all 1811 randomized trial participants, 465 patients (25.7 %) were treated for at least one bifurcated lesion. Patients with bifurcated lesions were predominantly men, presented more often with stable angina, and underwent more often treatment of multiple vessels and lesions in left anterior descending arteries (Tables 1, 2). Most patients (83.2 %) were treated with single stents. If a two-stent technique was applied, T-stenting (73.1 %) was generally preferred above (mini-)crush (17.9 %), culotte (2.6 %), and other two-stent approaches (6.4 %) (Table 2). Final kissing-balloon inflation was performed in 139 (29.9 %) patients with bifurcated target lesions.

**Table 1:** Patient characteristics of all study patients comparing patients with bifurcated versus non-bifurcated target lesions

Patient characteristics	All patients *n* = 1811		*p*
	BL *n* = 465	Non-BL *n* = 1346	
Age (years)	63.8 ± 11.3	64.0 ± 10.7	0.74
Female	100 (21.5)	389 (28.9)	0.002
BMI (kg/m^2^)	27.9 ± 4.5*	28.0 ± 4.8^†^	0.71
Diabetes mellitus	83 (17.8)	241 (17.9)	0.98
Previous MI	112 (24.1)	285 (21.2)	0.19
Previous PCI	85 (18.3)	264 (19.6)	0.53
Previous CABG	42 (9.0)	131 (9.7)	0.66
Clinical syndrome at index PCI procedure			
Stable angina pectoris	218 (46.9)	531 (39.5)	0.005
Unstable angina pectoris	57 (12.3)	188 (14.0)	
Non-ST-elevation myocardial infarction	118 (25.4)	329 (24.4)	
ST-elevation myocardial infarction	72 (15.5)	298 (22.1)	

Values are mean ± SD or *n* (%)

*BL* bifurcated target lesion, *CABG* coronary artery bypass grafting, *MI* myocardial infarction, *non-BL* non-bifurcated target lesion, *PCI* percutaneous coronary intervention

* *n* = 375, ^†^ *n* = 1049

**Table 2:** Lesion and procedural characteristics of all study patients comparing patients with bifurcated versus non-bifurcated target lesions

Lesion/procedural characteristics	All patients (*n* = 1811)		*p*
	BL *n* = 465	Non-BL *n* = 1346	
Multivessel treatment	123 (26.5)	173 (12.9)	<0.001
Treated coronary vessels			
Right coronary artery	91 (19.6)	578 (42.9)	<0.001
Left anterior descending artery	336 (72.3)	518 (38.5)	<0.001
Circumflex artery	148 (31.8)	375 (27.9)	0.10
De novo lesion	423 (91.0)	1204 (89.5)	0.35
Severe calcification	106 (22.8)	301 (22.4)	0.85
At least one chronic total occlusion	19 (4.1)	57 (4.2)	0.89
At least one in-stent restenosis	17 (3.7)	38 (2.8)	0.37
At least one small vessel	298 (64.1)	770 (57.2)	0.01
At least one lesion length >27 mm	95 (20.4)	223 (16.6)	0.06
Medina classification of bifurcation lesions			
0.0.1	37 (8.0)		
0.1.0	67 (14.4)		
0.1.1	21 (4.5)		
1.0.0	55 (11.8)		
1.0.1	39 (8.4)		
1.1.0	148 (31.8)		
1.1.1	98 (21.1)		
Total stent length	36.0 (22.0–56.0)	28.0 (18.0–48.0)	<0.001
Number of stents per patient	2.1 (1.3)	1.7 (1.0)	<0.001
Longest lesion length (mm)	19.4 (12.0)	18.3 (12.1)	0.09
Degree of stenosis (pre-PCI)*	70.2 (16.9)	71.1 (18.2)	0.36
Residual in-stent stenosis (post-PCI)*	17.9 (8.7)	17.3 (8.1)	0.22
Postdilation	397 (85.4)	1006 (74.7)	<0.001
Stenting approach			
1-stent	387 (83.2)		
2-stent	78 (16.8)		
T-stenting	57 (73.1)		
(Mini)crush	14 (17.9)		
Culotte	2 (2.6)		
Other	5 (6.4)		
Final kissing-balloon inflation	139 (29.9)		

Values are mean ± SD, *n* (%), or median (IQR)

*BL* bifurcated target lesion, *non-BL* non-bifurcated target lesion, *PCI* percutaneous coronary intervention

* A reference vessel diameter of <2.75 mm is defined as a small vessel

^†^In the case of multiple target lesions, the most severe diameter stenosis is presented

Among all patients with bifurcated lesions, 244 (52.5 %) were treated with resolute integrity and 221 (47.5 %) with promus element stents. The characteristics of patients, lesions, and procedures did not differ between the two stent groups (data not shown) except for a higher rate of kissing-balloon inflation in resolute integrity stents (36.1 versus 23.1 %; *p* = 0.002).

### Clinical event rates and multivariate analysis at 2-year follow-up

**Fig. 1:**
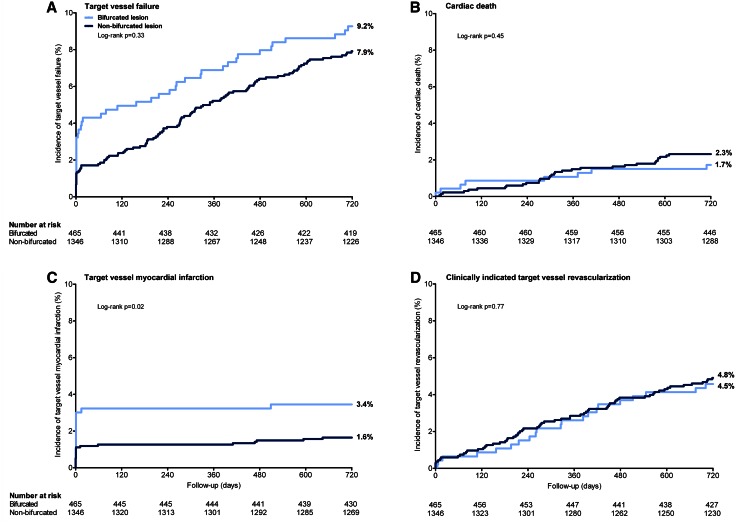
Kaplan–Meier curves of the composite clinical endpoint target vessel failure and its individual components: **a** target vessel failure; **b** cardiac death; **c** target vessel revascularization; **d** clinically indicated target vessel revascularization

Two-year follow-up data were available for 1810 (99.9 %) patients. Time-to-event analysis of patients with bifurcated lesions and patients with non-bifurcated lesions showed no significant differences in the rates of target vessel failure (9.2 versus 7.9 %; *p* logrank = 0.33), cardiac death (1.7 versus 2.3 %; *p* logrank = 0.45), and target vessel revascularization (4.5 versus 4.8 %; *p* logrank = 0.77) (Fig. 1). Target vessel MI was higher in patients with bifurcation lesions (3.4 versus 1.6 %; *p* logrank = 0.02) (Fig. 1; Table 3), but after multivariate analysis with propensity score adjustment, bifurcation treatment was found not to be an independent predictor of target vessel MI (HR 1.40, 95 % CI 0.71–2.76; *p* = 0.34). The rates of definite stent thrombosis after 2 years were low and comparable for both patients with bifurcated and non-bifurcated lesions (0.4 versus 1.0 %; *p* = 0.38).

**Table 3:** Two-year clinical outcome in patients with bifurcated lesions versus non-bifurcated lesions

	All patients *n* = 1810		*p*
	BL *n* = 465	Non-BL *n* = 1345	
Cardiac death	8 (1.7)	31 (2.3)	0.45
Target vessel myocardial infarction	16 (3.4)	22 (1.6)	0.02
Periprocedural myocardial infarction*	15 (3.2)	15 (1.1)	0.002
Target vessel revascularization^†^	21 (4.5)	65 (4.8)	0.78
Target lesion revascularization^†^	16 (3.4)	50 (3.7)	0.78
Definite stent thrombosis	2 (0.4)	13 (1.0)	0.38
Definite or probable stent thrombosis	4 (0.9)	16 (1.2)	0.56
Target vessel failure	43 (9.2)	106 (7.9)	0.36
Target lesion failure	38 (8.2)	93 (6.9)	0.37
Major adverse cardiac events	43 (9.2)	112 (8.3)	0.54
Patient-oriented composite endpoint	60 (12.9)	168 (12.5)	0.82

Values are *n* (%)

Two-year follow-up was available for 1810 of 1811 patients (99.9 %)

*BL* bifurcated target lesion, *Non-BL* non-bifurcated target lesion

* Periprocedural myocardial infarction is a sub-classification of (any) target vessel myocardial infarction; ^†^ clinically indicated

### Clinical outcome among patients with bifurcated lesions

**Fig. 2:**
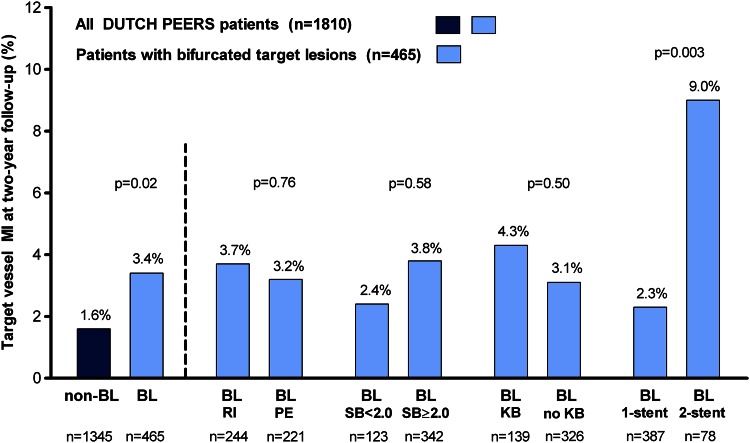
Target vessel MI rate at 2-year follow-up. *BL* bifurcated target lesion, *KB* final kissing-balloon inflation, *MI* myocardial infarction, *PE* promus element, *RI* resolute integrity, *SB* side-branch. Two-year follow-up was available for 1810 of 1811 patients (99.9 %)

Among patients with bifurcated target lesions, the rates of various clinical endpoints were similar for patients treated with resolute integrity versus promus element stents (Table 4). There was also no significant difference in any clinical endpoint between bifurcated lesions with side-branch ≥2.0 versus <2.0 mm, and between the use of final kissing-balloon inflation versus no use of final kissing balloons (Table 4; Fig. 2). The use of a two-stent approach resulted in significantly higher PMI rates than the use of a single stent (9.0 versus 2.1 %; *p* = 0.002).

**Table 4:** Two-year clinical outcome in patients among patients treated for bifurcated lesions

Patient characteristics	Stent used *n* = 465			Maximum side-branch (SB) diameter (mm) *n* = 465			Kissing-balloon inflation *n* = 465			Stenting approach *n* = 465		
	Resolute integrity *n* = 244	Promus element *n* = 221	*p*	SB < 2.0 *n* = 123	SB ≥ 2.0 *n* = 342	*p*	KB *n* = 139	No KB *n* = 326	*p*	1-Stent *n* = 387	2-Stent *n* = 78	*p*
Cardiac death	4 (1.6)	4 (1.8)	1.00	0	8 (2.3)	0.12	1 (0.7)	7 (2.1)	0.45	7 (1.8)	1 (1.3)	1.00
Target vessel myocardial infarction	9 (3.7)	7 (3.2)	0.76	3 (2.4)	13 (3.8)	0.58	6 (4.3)	10 (3.1)	0.50	9 (2.3)	7 (9.0)	0.003
Periprocedural myocardial infarction*	9 (3.7)	6 (2.7)	0.55	3 (2.4)	12 (3.5)	0.57	6 (4.3)	9 (2.8)	0.39	8 (2.1)	7 (9.0)	0.002
Target vessel revascularization^†^	12 (4.9)	9 (4.1)	0.66	7 (5.7)	14 (4.1)	0.46	4 (2.9)	17 (5.2)	0.27	5 (3.6)	17 (5.2)	0.45
Target lesion revascularization^†^	10 (4.1)	6 (2.7)	0.41	7 (5.7)	9 (2.6)	0.11	4 (2.9)	12 (3.7)	0.88	15 (3.9)	2 (0.5)	0.75
Definite stent thrombosis	1 (0.4)	1 (0.5)	1.00	0	2 (0.6)	1.00	0	2 (0.6)	1.00	2 (0.5)	0	1.00
Definite or probable stent thrombosis	2 (0.8)	2 (0.9)	1.00	0	4 (1.2)	0.58	1 (0.7)	3 (0.9)	1.00	3 (0.8)	1 (1.3)	0.52
Target vessel failure	24 (9.8)	19 (8.6)	0.65	10 (8.1)	33 (9.6)	0.62	11 (7.9)	32 (9.8)	0.52	32 (8.3)	11 (14.1)	0.11
Target lesion failure	22 (9.0)	16 (7.2)	0.49	10 (8.1)	28 (8.2)	0.98	11 (7.9)	27 (8.3)	0.89	28 (7.2)	10 (12.8)	0.10
Major adverse cardiac events	24 (9.8)	19 (8.6)	0.65	10 (8.1)	33 (9.6)	0.62	12 (8.6)	31 (9.5)	0.77	32 (8.3)	11 (14.1)	0.11
Patient-oriented composite endpoint	33 (13.5)	27 (12.2)	0.68	12 (9.8)	48 (14.0)	0.23	13 (9.4)	47 (14.4)	0.14	47 (12.1)	13 (16.7)	0.28

Values are *n* (%)

*KB* final kissing-balloon inflation, *SB* side-branch

* Periprocedural myocardial infarction is a sub-classification of (any) target vessel myocardial infarction; ^†^ clinically indicated

## Discussion

### Major findings

In the present subanalysis of the DUTCH PEERS randomized trial, all-comer patients treated for at least one bifurcated lesion versus patients with non-bifurcated target lesions showed similar 2-year rates of various clinical endpoints. Target vessel MI was more common in patients with bifurcation lesions (3.4 versus 1.6 %); but after multivariate analysis, bifurcation treatment was found not to be an independent predictor of target vessel MI. Among patients with bifurcated lesions, we found no impact of DES type, side-branch size, or final kissing-balloon inflation on various clinical endpoints. PMI was more common among patients with bifurcation lesions treated with a two-stent technique. Our findings suggest that the novel, flexible DES used are efficacious and safe for treating bifurcated target lesions.

### Stent design and outcome of PCI in bifurcated lesions

Previous studies with second-generation DES examined devices with the same drug-eluting coatings, but rather different designs of the stent platforms, as used in the devices of the present study [20, 21]. In a subanalysis of the TWENTE trial, patients treated for bifurcation lesions with second-generation Resolute (Medtronic) or Xience V stents (Abbott Vascular Devices, Santa Clara, CA) showed favorable 3-year outcomes that were similar to the outcomes in patients with non-bifurcated lesions (e.g., target vessel failure rate 13.1 versus 12.6 %), but in patients with bifurcated lesions the PMI rate was more than twice as high (6.9 versus 3.1 %; *p* < 0.01) [20]. These data corroborated the results of a substudy of the RESOLUTE All Comers trial, which had also reported a higher PMI rate in 392 patients with bifurcated lesions (6.5 versus 3.4 %; unadjusted *p* = 0.009) [21]. In a pooled analysis of the RESOLUTE All Comer trial and the RESOLUTE International Registry, the incidence of target lesion failure and the individual components thereof was higher during the first 30 days after PCI of patients who were treated for bifurcation lesions as compared to patients treated for non-bifurcated lesions. However, during the remainder of the 3-year follow-up, clinical event rates were similar for both patient groups [29]. PMI in treatment of bifurcated lesions may result from (stent-induced) closure of side-branches, flow-limiting dissections, distal (micro)embolization of atherothrombotic debris, and the occurrence of slow flow or no-reflow [20, 30].

The development of newer-generation DES and the progression into devices with highly flexible stent platforms have reduced the need for repeat revascularization and the rate of target vessel MI following PCI of bifurcated lesions [3–9, 11, 19–22, 31]. Burzotta et al. used virtual bench tests to assess the impact of technical characteristics of DES platforms on stenting in bifurcated lesions, showing that technical features of DES platforms lead to differences in response to similar procedural steps of provisional stenting, such as final kissing-balloon inflation [23]. Therefore, technical characteristics of stents should be taken into account in the selection process of the most appropriate DES for treatment of bifurcated lesions [23].

Both resolute integrity and promus element stents have demonstrated favorable results in the all-comer patient population of the DUTCH PEERS randomized trial [24]. The present study of patients with bifurcated lesions has shown similar rates of various clinical endpoints for both stent groups. The baseline characteristics of patients with bifurcated lesions in both DES arms were comparable, but in promus element stents final kissing-balloon inflation was less often performed. As the actual motives of the operators were not documented in this context, we can only speculate that knowledge about the somewhat increased risk of longitudinal deformation of the promus element stent [22] might have held some operators back from performing final simultaneous kissing-balloon inflations.

### Final kissing-balloon inflation

The potential impact of a final kissing-balloon inflation on clinical outcome following stenting of bifurcated lesions is still unclear [20, 32–34]. Niemelä et al. investigated the use of routine final kissing-balloon inflation after successful stenting of the main branch with a single stent. Despite a reduced rate of angiographic side-branch (re)stenosis following kissing-balloon inflation, clinical outcome (PMI was not included) was similar for patients treated with versus without final kissing-balloon inflation [32]. Three-year outcome data of the TWENTE trial have also shown similar target vessel failure rates in patients with bifurcated lesions who were treated with or without final kissing-balloon inflation [20], while final kissing-balloon inflation was reported to be beneficial following treatment of true bifurcation lesions with single, predominantly first-generation DES in patients with acute coronary syndromes [34]. It is likely that the inconsistent results of final kissing-balloon inflation with different stent types are caused by differences in the specific technical stent characteristics, leading to different stent strut distributions after final kissing-balloon inflation [23].

### Side-branch size

Previous studies of stenting in bifurcations used different criteria to define relevant side-branches and studied dissimilar patient populations, which renders comparison of their event rates difficult [3, 8, 19, 21, 32, 35]. In contrast to several other trials that considered side-branches ≥1.75 mm [4], ≥2.0 mm [5, 19, 33, 34, 36–38], ≥2.25 mm [31, 32, 35], or ≥2.5 mm [3] as relevant, the present study defined side-branches to be relevant if they had a minimum lumen diameter ≥1.5 mm by quantitative coronary angiography, as suggested by the investigators of the SYNTAX trial [26]. Nevertheless, when comparing clinical outcome of patients with bifurcated lesions and side-branches <2.0 mm versus side-branches ≥2.0 mm, we found no relation between the side-branch size and the risk of various clinical endpoints including target vessel MI.

### Single versus two-stent approach

Previous studies that compared the outcome of bifurcation treatment with two-stent strategies versus the use of a single stent suggested more often, similar to the results of the present substudy, a higher risk of PMI following two-stent procedures [3, 21, 29, 35, 39]. It has been speculated that during the more complex two-stent procedures the longer duration of vessel instrumentation, the more frequent balloon and stent passages through vessel segments proximal to the bifurcation, and the generally higher frequency of stent postdilation may contribute to the higher PMI risk [35]. A slight disadvantage of the single-stent approach may be the somewhat higher risk of side-branch occlusion after stenting the main branch [37, 40]. Predictors of side-branch occlusion are: a high pre-procedural degree of side-branch stenosis; a calcified side-branch lesion; a long obstructed side-branch segment; proximal disease in the main branch; and treatment for an acute coronary syndrome [40]. In such bifurcation lesions with an increased risk of jeopardizing the side-branch, the straightforward use of a two-stent technique will often increase the likelihood of keeping the side-branch patent [41].

### Limitations

Because of the post hoc nature of the present analysis, the results must be considered hypothesis generating. Nevertheless, in the absence of published data on PCI in bifurcated lesions with these novel, flexible DES, the findings may be of interest. Similar to previous studies [20, 21], the sample size of subgroups among patients with bifurcated lesions was limited. Therefore, the results of subgroup analyses should be interpreted with caution.

## Conclusion

All-comer patients treated for bifurcated and non-bifurcated target lesions showed similar and low rates of clinical endpoints, suggesting that the DES used are efficacious and safe for treating bifurcated target lesions.

## Abbreviations

DES: Drug-eluting stent
MI: Myocardial infarction
PCI: Percutaneous coronary intervention
PMI: Periprocedural MI
